# Supplementary material for: FERN – a Java framework for stochastic simulation and evaluation of reaction networks
Source: BMC Bioinformatics. 2008 Aug 29;9:356. doi: 10.1186/1471-2105-9-356 (PMC2553347; doi:10.1186/1471-2105-9-356)
Supplement: Additional file 1 — FERN distribution, Version 1.3. This archive contains the FERN source code and binaries as well as documentation and example models in FernML and SBML. [file 1471-2105-9-356-S1.zip › fern/doc/javadoc/fern/simulation/observer/AmountAtMomentObserver.html]

AmountAtMomentObserver


---


|  |  |  |
| --- | --- | --- |
| **Overview** \| **Package** \| **Class** \| **Use** \| **Tree** \| **Deprecated** \| **Index** \| **Help** | |  |
| PREV CLASS   **NEXT CLASS** | **FRAMES**    **NO FRAMES**     **All Classes** |  |
| SUMMARY: NESTED \| FIELD \| CONSTR \| METHOD | DETAIL: FIELD \| CONSTR \| METHOD |  |


---


## fern.simulation.observer Class AmountAtMomentObserver

```
java.lang.Object
  fern.simulation.observer.Observer
      fern.simulation.observer.AmountAtMomentObserver
```

**All Implemented Interfaces:**: SimulationController, GnuPlotObserver

---

``` public class AmountAtMomentObserver extends Observer implements SimulationController, GnuPlotObserver ```

Observes the amount of some molecule species at a certain moment. In order to
accomplish that, this class also implements a `SimulationController` and
registers a `theta` at the simulator. The data produced after are histograms
for each species representing the distribution of amounts at theta.

**Author:**
:   Florian Erhard

**See Also:**: `Observer.getTheta()`

---

**Constructor Summary**

`AmountAtMomentObserver(Simulator sim,
double moment,
String... speciesName)`
  
          Creates the observer for the given simulator, the given theta and the given species


| **Method Summary** | |
| --- | --- |
| `void` | `activateReaction(int mu, double tau, Simulator.FireType fireType, int times)`             Do nothing |
| `void` | `finished()`             Do nothing. |
| `Map<Integer,Integer>` | `getHistogram(int species)`             Gets the histogram of the distribution for a species |
| `String[]` | `getStyles()`             Gets the styles for the columns. |
| `boolean` | `goOn(Simulator sim)`             Causes the simulator to stop when `theta` has been passed and the amounts have been recorded. |
| `void` | `started()`             Register theta at the simulator. |
| `void` | `step()`             Do nothing. |
| `void` | `theta(double theta)`             Records the amounts of the species and puts it into the histogram. |
| `GnuPlot` | `toGnuplot()`             Creates a new `GnuPlot` object and passes the actual observer data to it. |
| `GnuPlot` | `toGnuplot(GnuPlot gnuplot)`             Passes the actual observer data to a `GnuPlot` object. |
| `String` | `toString()` |

**Methods inherited from class fern.simulation.observer.Observer**

`applyLabelFormat, getLabel, getLabelFormat, getNumSimulations, getPrintWriter, getSimulator, getTheta, getTitlesCommand, print, setLabelFormat, setPrintWriter, setSimulator, setTheta`

**Methods inherited from class java.lang.Object**

`clone, equals, finalize, getClass, hashCode, notify, notifyAll, wait, wait, wait`

**Constructor Detail**

### AmountAtMomentObserver

```
public AmountAtMomentObserver(Simulator sim,
                              double moment,
                              String... speciesName)
```

:   Creates the observer for the given simulator, the given theta and the given species

    **Parameters:**: `sim` - simulator: `moment` - theta: `speciesName` - names of the species to observe


**Method Detail**

### goOn

```
public boolean goOn(Simulator sim)
```

:   Causes the simulator to stop when `theta` has been passed and the amounts
    have been recorded.

    :   **Specified by:**: `goOn` in interface `SimulationController`
    :   **Parameters:**: `sim` - simulation **Returns:**: whether or not to go on

---


### activateReaction

```
public void activateReaction(int mu,
                             double tau,
                             Simulator.FireType fireType,
                             int times)
```

:   Do nothing

    :   **Specified by:**: `activateReaction` in class `Observer`
    :   **Parameters:**: `mu` - the reaction which is supposed to fire: `tau` - the time the reaction fires (at this time `Simulator.getTime()` does not necessarily yield the firing time): `fireType` - the type of the firing: `times` - TODO

---


### theta

```
public void theta(double theta)
```

:   Records the amounts of the species and puts it into the histogram.

    :   **Specified by:**: `theta` in class `Observer`
    :   **Parameters:**: `theta` - moment in time

---


### finished

```
public void finished()
```

:   Do nothing.

    :   **Specified by:**: `finished` in class `Observer`

---


### started

```
public void started()
```

:   Register theta at the simulator.

    :   **Specified by:**: `started` in class `Observer`

---


### step

```
public void step()
```

:   Do nothing.

    :   **Specified by:**: `step` in class `Observer`

---


### toGnuplot

```
public GnuPlot toGnuplot()
                  throws IOException
```

:   **Description copied from interface: `GnuPlotObserver`**
:   Creates a new `GnuPlot` object and passes the actual observer data to it.

    :   **Specified by:**: `toGnuplot` in interface `GnuPlotObserver`
    :   **Returns:**: the created `GnuPlot` object **Throws:**: `IOException` - if gnuplot could not be accessed

---


### toGnuplot

```
public GnuPlot toGnuplot(GnuPlot gnuplot)
                  throws IOException
```

:   **Description copied from interface: `GnuPlotObserver`**
:   Passes the actual observer data to a `GnuPlot` object.

    :   **Specified by:**: `toGnuplot` in interface `GnuPlotObserver`
    :   **Parameters:**: `gnuplot` - the `GnuPlot` object to pass the data to **Returns:**: the `GnuPlot` object **Throws:**: `IOException` - if gnuplot could not be accessed

---


### getHistogram

```
public Map<Integer,Integer> getHistogram(int species)
```

:   Gets the histogram of the distribution for a species

    :   **Parameters:**: `species` - species index (but not in network index space - i means the ith passed species) **Returns:**: histogram as map

---


### toString

```
public String toString()
```

:   **Overrides:**: `toString` in class `Object`

---


### getStyles

```
public String[] getStyles()
```

:   **Description copied from interface: `GnuPlotObserver`**
:   Gets the styles for the columns. If you don't want styles, just return null!

    :   **Specified by:**: `getStyles` in interface `GnuPlotObserver`
    :   **Returns:**: styles for the columns


---


|  |  |  |
| --- | --- | --- |
| **Overview** \| **Package** \| **Class** \| **Use** \| **Tree** \| **Deprecated** \| **Index** \| **Help** | |  |
| PREV CLASS   **NEXT CLASS** | **FRAMES**    **NO FRAMES**     **All Classes** |  |
| SUMMARY: NESTED \| FIELD \| CONSTR \| METHOD | DETAIL: FIELD \| CONSTR \| METHOD |  |


---
